# Postnatal Calyceal-to-Parenchymal Ratio: A Promising Predictor for Surgical Correction of Ureteropelvic Junction Obstruction in Newborns

**DOI:** 10.7759/cureus.48466

**Published:** 2023-11-07

**Authors:** Fayez Almodhen, Wael M Moneir, Ameen Bashareef, Ahmed Al-Zahrani, Abdullah Alaqeel, Abdulwahab Alhams, Yasser Jamalalail, Tariq Burki, Ahmed AlShammari

**Affiliations:** 1 Department of Pediatric Urology, King Abdullah Specialized Children's Hospital, King Abdulaziz Medical City, Ministry of National Guard Health Affairs, Riyadh, SAU; 2 Department of Urology, Ali Bin Ali Hospital, Riyadh, SAU; 3 Department of Medical Imaging, Pediatric Radiology Section, King Abdullah Specialized Children's Hospital, King Abdulaziz Medical City, Ministry of National Guard Health Affairs, Riyadh, SAU

**Keywords:** ureteropelvic junction obstruction, pyeloplasty, predictor, hydronephrosis, calyceal-to-parenchymal ratio, antenatal hydronephrosis

## Abstract

Objective: This study aims to explore a new parameter, the calyceal-to-parenchymal ratio (CPR) of postnatal renal ultrasonography (RUS) as a predictor of surgery in newborns with possible ureteropelvic junction obstruction (UPJO). Although UPJO remains the main surgical category of antenatally detected hydronephrosis, there is a lack of a gold-standard test that predicts the need for pyeloplasty.

Subjects and methods: We retrospectively reviewed infants with a positive antenatal history of hydronephrosis who were confirmed to have grade 3 or 4 hydronephrosis on postnatal RUS between 2010 and 2020. We compared postnatal CPR between surgical and control groups and tested the correlation between postnatal CPR and diuretic renogram.

Results: A total of 79 and 136 kidneys were included in the surgical and control groups, respectively. Kidneys that were managed with pyeloplasty between January 2010 and July 2020 were included in the surgical group, while kidneys from patients with comparable traits who were managed conservatively comprised the control group. At a mean age of 18.9 weeks at presentation and a mean follow-up period of 48.99 months, the median postnatal CPR was significantly greater in the surgical group (3.62 vs. 0.98, p<0.001). A postnatal CPR of 1.68 had a sensitivity and specificity of 96.2% and 84.8%, respectively, in predicting the need for future pyeloplasty (area under the curve (AUC)=0.966). There was a positive and significant correlation between postnatal CPR and the half-life of the renogram (p=0.018) but not significant with the differential function (p=0.090).

Conclusion: Diuretic renography has little capability for predicting future pyeloplasty. Current RUS grading systems do not offer an objective measure of renal parenchyma. Numerous other RUS parameters are less frequently utilized in clinical practice, and many are challenging to assess and require sophisticated software or equipment.

Postnatal CPR is a promising tool for predicting the need for pyeloplasty in newborns with UPJO. Further prospective studies are needed to standardize and assess the reproducibility of this parameter.

## Introduction

With the introduction of prenatal ultrasonography, antenatally detected hydronephrosis is seen in approximately 1-5% of all prenatal scans [1]. Ureteropelvic junction obstruction (UPJO) represents one of the most common surgical entities associated with antenatal hydronephrosis [2]. Despite the increased incidence of antenatally diagnosed urinary tract dilatation, the distinction between urinary tract obstruction and non-obstructive urinary tract dilatation remains a challenge [3]. The indefinite nature of the currently available diagnostic means left the widely recognized definition of UPJO an annoying retrospective diagnosis based on significant worsening hydronephrosis on serial RUS and/or a drop in the differential function of the kidney [4]. Working with this definition, many guidelines to investigate and manage UPJO have been established; however, indications for surgery will always be disputable since the critical goal is to determine which child should undergo pyeloplasty and which child could be managed conservatively with careful follow-up [5].

Postnatal evaluation of hydronephrosis is mostly dependent on renal ultrasonography (RUS) and diuretic renography [6]. Many studies are currently focusing on the prediction values of RUS parameters, not only because RUS will avoid the risk of ionizing radiation associated with renography but also because of its non-invasive nature, feasibility, and cost savings [6-8]. Unfortunately, the most widely recognized RUS systems to evaluate hydronephrosis, including the Society of Fetal Urology (SFU) hydronephrosis grading system and the anterior-posterior renal pelvic diameter (APRPD), do not come without major pitfalls, and the SFU grading system is subjective, unable to distinguish diffuse and segmental parenchymal thinning, and fails to demonstrate accurately the severity of hydronephrosis in higher grades [7]. APRPD may differ depending on the hydration status of the patient, bladder volume, and position of the patient during the scan [9].

A robust search for alternative RUS parameters for evaluation and follow-up of postnatal hydronephrosis has led to a novel approach to evaluating the kidney using sonographic renal parenchymal measurements [10]. Since UPJO leads to hydronephrosis, parenchymal atrophy, and eventually impaired renal function, recent studies have highlighted a new parameter, calyceal-to-parenchymal ratio (CPR), in the evaluation and follow-up of patients treated with pyeloplasty as a predictor of successful intervention [11,12].

In this study, we aimed to assess a new approach using the CPR of postnatal RUS as an initial predictor for pyeloplasty. The renal parenchyma will directly suffer from an increase in intra-calyceal pressures secondary to obstruction. We hypothesize that CPR can be used to evaluate the future need for pyeloplasty. We assessed CPR as a predictor of pyeloplasty compared to diuretic renography. Our study has tested a potential measurement to avoid the harmful effects of UPJO on the kidney.

## Materials and methods

This study was carried out by King Abdullah Specialized Children's Hospital, Riyadh, Saudi Arabia, from January 2010 to July 2020. The Ethics Committee of King Abdullah International Medical Research Centre, King Saud bin Abdulaziz University for Health Sciences, Riyadh, Saudi Arabia, approved the study (approval number: H-01-R-005, approval date: 24/08/2020). We retrospectively reviewed the charts of infants who had been born in our institute or referred to us with a positive antenatal history of hydronephrosis and were confirmed to have SFU grade 3 or 4 hydronephrosis on the postnatal scan.

The early workup included postnatal RUS in the first six weeks, but not within the first 72 hours of life, to prove the antenatal diagnosis. All patients were also evaluated with a voiding cystourethrogram within the first weeks of life. Patients with low-grade hydronephrosis (SFU grade 1 or 2), associated vesicoureteral reflux, ureterovesical junction obstruction, posterior urethral valve, and vesical or other infra-vesical obstructing pathologies were excluded.

A standard diuretic renogram (mercaptoacetyltriglycine-3, MAG-3) was postponed until four to six weeks of age, and the differential renal function (DRF) of the affected kidney and the drainage half-time (T1/2) in the post-furosemide renogram curve were recorded. The T1/2 was considered obstructed if it was more than 20 minutes, non-obstructed if it was less than 10 minutes, and equivocal if it was between 10 and 20 minutes.

Kidneys with a function of less than 40% proceeded to pyeloplasty, while the remaining kidneys were re-investigated with RUS and diuretic renography at three months, six months, one year, and then once a year till the age of five years. The diuretic renography was ignored if there was a continuous improvement in the degree of hydronephrosis during the follow-up. Kidneys that were managed with pyeloplasty during the study period were included in the surgical group, while kidneys from patients with comparable traits who were managed conservatively comprised the control group. Indications for surgical intervention in our surgical group were worsening hydronephrosis on serial RUS, less than 40% DRF on the initial renogram, or a drop in DRF by more than 10%, and clinically symptomatic patients. All included patients were taking a daily dose of co-trimoxazole prophylaxis (2 mg/kg at night) for the first year of life.

**Figure 1:**
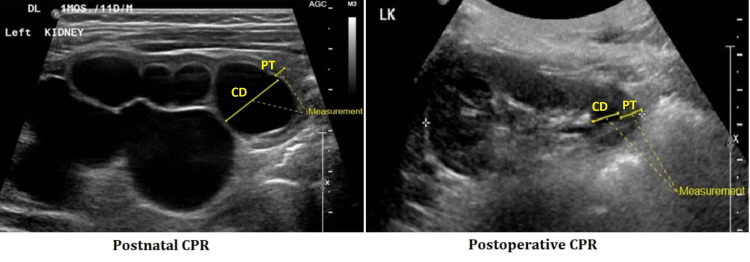
Measuring the CPR Sagittal ultrasonographic image of hydronephrotic kidney representing the measurement of CD and PT to calculate postnatal CPR and CPR after pyeloplasty for the same patient CPR: calyceal-to-parenchymal ratio, CD: calyceal diameter, PT: parenchymal thickness

For the measurement of the CPR, the most comparable and representative sagittal plane views on the RUS scans were used. The calyx with the maximum dilatation, usually a polar calyx, was selected for calyceal diameter (CD), and the corresponding parenchymal thickness (PT), in the same line as the measured CD, was measured. The ratio was calculated by dividing the CD/PT (Figure 1).

Data recorded during the study included date of birth, sex, affected kidney, degree of hydronephrosis, CPR of the affected kidney in different RUS scans (postnatal scan, postoperative scan for surgical patients, and the RUS with downgrading of the degree of hydronephrosis for conservative cases), and renogram findings (DRF and T1/2). All radiological investigations were interpreted by a single, expert pediatric radiologist.

Data was collected using an MS Excel spreadsheet (Microsoft 365, Microsoft Corp., Redmond, WA, USA). The statistical analysis was carried out using IBM SPSS Statistics for Windows, version 21 (IBM Corp., Armonk, N.Y., USA). Descriptive statistics, including mean, median, standard deviation, and interquartile range (IQR), were measured. The Kolmogorov-Smirnov-Smirnov normality was used to check the normality of the data. Categorical variables were compared using Chi-square. The Mann-Whitney test was used to compare the postnatal CPR in the two groups. The area under the receiver operating characteristic (ROC) curve was calculated to define a cut-off point in postnatal CPR that separates the surgical group and the controls. In the surgical group, postnatal and postoperative CPR were compared using the Wilcoxon signed-rank test. Correlations between postnatal CPR and renogram were tested using Spearman's test. All statistical tests were performed at a significance level of α=0.05 and were two-sided.

## Results

A total of 376 consecutive kidneys from 294 patients with SFU grades 3 and 4 in the period between January 2010 and July 2020 were screened. One hundred sixty-one kidneys were excluded due to the presence of associated pathologies (vesicoureteral reflux (n=74), posterior urethral valve (n=33), ureterovesical junction obstruction (n=34), duplex kidney (n=11), and lost follow-up (n=9)).

Seventy-nine kidneys underwent pyeloplasty during the study period; the average age at surgery was 5.8 months. One hundred thirty-six consecutive kidneys from patients who were managed conservatively were included in the control group. SFU grade 4 affected 61 and 47 kidneys in the surgical and control groups, respectively (p<0.001). The age of patients at presentation ranged from zero days to 18.9 weeks (1.88 ± 3.82 weeks). The mean follow-up period was 48.99 ± 39.14 months (range 3-152 months). Regarding gender distribution, 170 (79.1%) kidneys were from male patients. Hydronephrosis affected 130 (60.5%) left kidneys (Table 1).

**Table 1:** Patients' characteristics Characteristics of included kidneys and patients with hydronephrosis who underwent pyeloplasty vs. conservative follow-up SFU: Society of Fetal Urology

	Pyeloplasty (N=79)	Conservative (N=136)	p-value
Number of kidneys	79 (76 patients)	136 (117 patients)	
Mean age at presentation	2.9 weeks	2.2 weeks	p=0.143
Gender			p=0.845
Male	62	108	
Female	17	28	
Affected kidney			p=0.872
Left	47	83	
Right	32	53	
Grade of hydronephrosis			p<0.001
SFU 3	18	89	
SFU 4	61	47	

The median (IQR) CPR in the postnatal RUS of the surgical group was 3.62 (3.07), while the conservative group had a median (IQR) CPR of 0.98 (0.73). When comparing both groups, CPR of the postnatal RUS was significantly greater in the surgical group (p<0.001) (Table 2).

**Table 2:** Comparison of postnatal CPR Comparison of postnatal CPR in patients who underwent pyeloplasty vs. conservative follow-up IQR: interquartile range

	Pyeloplasty	Conservative	p-value
Median	3.62	0.98	p<0.001
IQR (Q3-Q1)	3.07 (5.52-2.45)	0.73 (1.43-0.69)	
Range	10.50	2.48	

**Figure 2:**
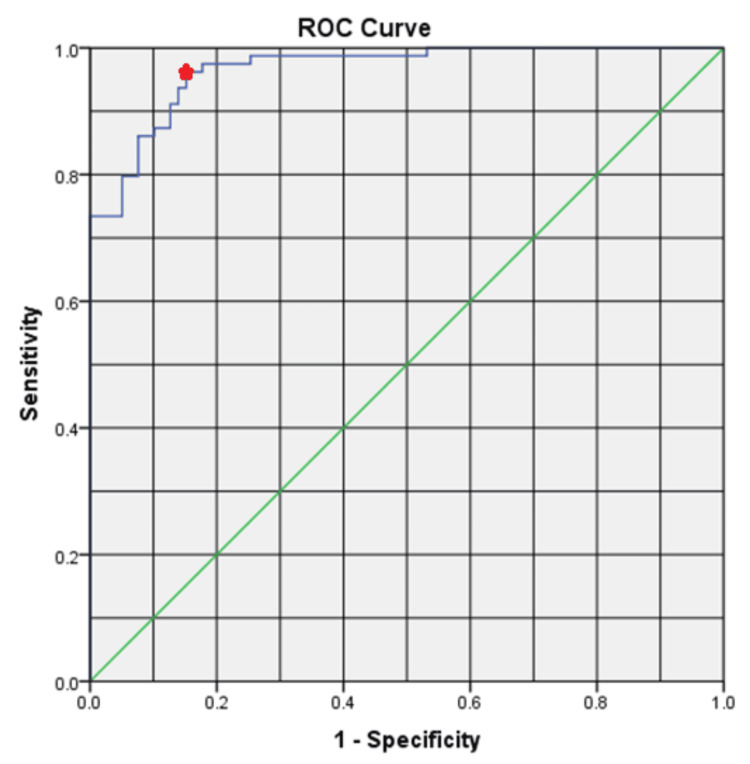
CPR predicting future pyeloplasty ROC curve analysis of postnatal CPR. The red point represents a CPR of 1.68 with a sensitivity and specificity of 96.2% and 84.8%, respectively, predicting the need for future pyeloplasty ROC: receiver operating characteristic

ROC curve was applied to define a cut-off point for CPR between the two groups. A CPR of 1.68 had a sensitivity and specificity of 96.2% and 84.8%, respectively, in predicting the need for pyeloplasty with an area under the curve (AUC) of 0.966 (Figure 2).

Spearman’s rank correlation was computed to assess the relationship between postnatal CPR and the preoperative renogram in the surgical group. There was a positive and significant correlation between postnatal CPR and the drainage half-life of the renogram (r=0.28, n=69, p=0.018). The T1/2 was considered obstructed if it lasted more than 20 minutes. The correlation between postnatal CPR and DRF was negative and insignificant (r=-0.21, n=69, p=0.090). On the other hand, the correlation between postnatal CPR and the degree of hydronephrosis in the postnatal RUS for the surgical group was positive and significant (r=0.50, n=79, p<0.001).

For the surgical group, a Wilcoxon signed-rank test indicated that postoperative CPR ranks were significantly lower than postnatal CPR ranks (z=-3.954, p<0.001, r=0.31).

## Discussion

Although UPJO is one of the most common presentations in pediatric urology, unfortunately, there is no gold-standard test for the diagnosis of obstruction. Diuretic renography is the most widely used investigation to evaluate renal function and drainage in newborns with suspected UPJO [6]. Unluckily, diuretic renography has little predictive capability in evaluating the need for future pyeloplasty [13], and it might seem paradoxical to wait until the function has declined and then perform pyeloplasty with the hopes of recovering the lost function [14]. Assmus et al. reported sensitivity and specificity of 0.61 and 0.56, respectively, for initial DRF, predicting a future ipsilateral DRF decline with an AUC of 0.561. DRF of the initial diuretic renography was a poor prognostic tool to detect who would suffer unfavorable outcomes (functional decline and pyeloplasty), as the discrimination ability of a test with an AUC of 0.561 is just a little better than chance [15]. Other studies have found no significant association between DRF and the likelihood of surgery [16], or as low as 11% association with DRF deterioration in patients followed conservatively [17]. Our results, on the other hand, demonstrated good sensitivity and specificity in predicting the need for later surgery. The insignificant correlation between postnatal CPR and DRF could be due to the timing of CPR measurement, the postnatal period, before the kidney has sustained the damage.

The data presented in this study have revealed that postnatal CPR is correlated with T1/2 of the diuretic renography. Although earlier studies have shown that the conventional classification of T1/2 may not be applicable to the evolving nature of UPJO in newborns [18], Krill et al. demonstrated that initial T1/2 on renography is predictive of future pyeloplasty [19]. Furthermore, Blum et al. proved that automated analysis of drainage curves from renography has potential clinical usefulness in the earlier detection of obstructed cases [18].

Alternatively, numerous RUS parameters to evaluate hydronephrosis with possible UPJO in children have been studied. The two most distinguished grading systems are the APRPD and the SFU. These systems have a drawback; both do not offer an objective measure of the renal parenchyma [8]. Moreover, a simple cut-off AP diameter value of the renal pelvis that separates obstructive from non-obstructive hydronephrosis of the kidney is not available [7]. UPJO is associated with increased intrapelvic pressure that leads to calyceal dilatation, parenchymal atrophy, and eventually impaired renal function [7,11]. Thus, postnatal CPR can be a good parameter to predict the need for pyeloplasty as early as possible. CPR incorporates PT and calyceal dilatation for the evaluation of the severity of hydronephrosis. PT is an objective parameter because, contrary to the pelvicalyceal system, it is not affected by hydration, bladder filling, or the patient’s position. A few years ago, it was reported that PT is a good predictor of postnatal pyeloplasty, especially if used as the APD/PT ratio [7,20]. Although calyceal dilatation might also be affected by hydration status and bladder volume, renal calyces have lower compliance and expandability when compared to the renal pelvis [7]. Moreover, CPR reflects intrapelvic pressure better than SFU and APRPD grades [11].

Recent studies assessed the rule of pyramidal thickness as a predictor for surgical intervention [21,22]. Despite the exciting results, pyramidal thickness, unlike CPR, can be difficult to assess because the medullary pyramid may easily disappear in severe hydronephrosis or loss of corticomedullary differentiation due to atrophy [10,21], and normative pyramidal thickness measurements in children with hydronephrosis are lacking [22].

Other parameters that have been studied include the parenchymal-to-pelvicalyceal area, pelvicalyceal area, calyx-to-parenchymal ratio, and pelvicalyceal volume on 3D RUS. It is frequently cited that these measurements are challenging to assess and require sophisticated software or equipment, making them less frequently utilized in clinical practice [8].

It is noteworthy that CPR was previously assessed for its utility in predicting the success and follow-up of pyeloplasty [11,12]. Our results support that CPR correlates well with postoperative hydronephrosis changes.

To the best of our knowledge, this is the first study to assess the prognostic value of postnatal CPR as a predictor of pyeloplasty. Postnatal CPR is easy to measure, does not require complicated software or specific equipment, and fulfills the crucial factor in categorizing hydronephrosis as a predictor for the need for surgical intervention.

Our study was not designed to replace renography and other US parameters in surgical decision-making. Our goal was simply to assess how postnatal CPR can predict the need for pyeloplasty. Postnatal CPR is a surveillance technique aimed at identifying, as early as possible, candidates for surgery to improve risk stratification. Our results also suggest that the burden of investigations needed during the follow-up period can be reduced.

One of the major limitations of our work is that it is a retrospective study. Prospective studies are needed to elucidate the reproducibility of the CPR and standardize the measurements during the routine postnatal RUS. The main limitation of this study is that indications of pyeloplasty are surgeon-dependent. A pediatric urologist can define his or her own criteria for pyeloplasty with some degree of deviation from guidelines. Using pyeloplasty as an outcome is another limitation of our study. Nevertheless, surgery is one of the few solid outcomes that are available in the UPJO natural history.

Despite the limitations, trying to predict which UPJO will need pyeloplasty is recommended. We urge pediatric urologists to adopt CPR in their practice to verify its prognostic capability and probably to establish an objective, simple measure that might aid in decision-making.

## Conclusions

To the best of our knowledge, this is the first study to explore a new parameter and has demonstrated that postnatal CPR is a promising tool as a predictor for the need for surgery in patients with UPJO that was detected antenatally. CPR is an easily measurable parameter that can be obtained from RUS without the need for complex software or machines, and it avoids the radiation hazards associated with diuretic renograms. Further prospective studies are needed to assess the reproducibility of this parameter.
